# Dietary Patterns in Children with Neurodevelopmental Disorders

**DOI:** 10.3390/nu16152460

**Published:** 2024-07-29

**Authors:** Laura Compañ-Gabucio, Laura Torres-Collado, Manuela García-de-la-Hera

**Affiliations:** 1 Unidad de Epidemiología de la Nutrición (EPINUT), Departamento de Salud Pública, Historia de la Ciencia y Ginecología, Universidad Miguel Hernández (UMH), 03550 Alicante, Spain; lcompan@umh.es (L.C.-G.); manoli@umh.es (M.G.-d.-l.-H.); 2 Instituto de Investigación Sanitaria y Biomédica de Alicante (ISABIAL), 03010 Alicante, Spain; 3 CIBER Epidemiología y Salud Pública (CIBERESP), Instituto de Salud Carlos III, 28034 Madrid, Spain

Neurodevelopmental disorders (NDDs), of which Autism Spectrum Disorder (ASD) and Attention Deficit Hyperactivity Disorder (ADHD) are two of the most common, are described as a group of conditions that begin in the developmental period and lead to deficits that impair functioning [1]. Children with NDDs experience multifaceted challenges, among which eating behavior and nutritional status require special attention. Eating disorders are mainly characterized by atypical eating behaviors, emotional and cognitive alterations, and dysregulated body weight. Children with NDDs, such as ASD or ADHD, often exhibit these atypical behaviors [2], possibly due to sensory processing alterations [3]. A previous scoping review [4] has summarized interventions for eating disorders in this population, although evidence in this area remains limited. This Special Issue provides a deeper insight into eating disorders in children with NDDs through two original studies and two review papers.

Vieira da Silva and Lopes Gomes [5] carried out a cross-sectional study with 80 children with ASD and their respective parents/caregivers in Brazil, a country with scarce scientific evidence on eating disorders in the pediatric population with NDDs. The results showed that the most prevalent eating behavior among children with ASD was food selectivity. In addition, these children presented a greater propensity to be overweight. These findings are consistent with previous scientific articles, which describe eating as a sensory experience involving a variety of smells, textures, appearances and tastes in foods [6]. Children with ASD also tend to have nutritional inadequacies that make them more vulnerable than their peers to developing obesity, overweight, and even underweight [2]. Vieira da Silva and Lopes Gomes emphasize that children with ASD are part of a nutritionally vulnerable group, and it is crucial to implement early interventions with personalized strategies to ensure treatment that is safe, complete, and appropriate for each child.

Thorsteinsdottir et al. [7] published an interesting intervention study providing a new perspective on eating disorders in children with NDDs. These researchers support the hypothesis that anxiety plays a significant role in the development and prevalence of eating disorders in this population. Children with NDDs appear to have higher levels of anxiety than their peers [8], and this can lead to disinhibited eating behaviors and feeding disturbances [9]. Although children with food selectivity experience anxiety when faced with foods they do not like [10], and despite the higher prevalence rates of anxiety in children with NDDs [8], there is a lack of research on anxiety and food selectivity in food-based interventions. In this sense, the authors conducted a Taste Education intervention involving 71 children with food selectivity to assess changes in anxiety between children with and without NDDs. The results indicated that this food-based intervention did not increase anxiety scores in children with food selectivity, regardless of their NDD status. Overall, they showed reductions in post-intervention aspects related to anxiety, such as physical symptoms and both social and separation anxiety. Therefore, this study proposed a valuable sensory-based, non-invasive, food-focused intervention for children with and without NDDs, which could lead to potential improvements in discomfort related to previously disliked foods. 

The review articles included in this Special Issue complement each other, so reading them both is highly recommended. Önal et al. [11] conducted a narrative review aimed at exploring the latest scientific findings on the role of diet in ASD. This narrative review, which followed a clear and easily replicable methodology, concludes that although there are numerous studies on dietary approaches in ASD, a clear recommendation for dietary intervention in this population cannot be made due to inconclusive evidence. Therefore, these results highlight the need for further research in order to enable an individualized dietary approach and to describe the role of dieticians in the therapeutic team. In addition, they underline the need for parents and caregivers of children with ASD to collaborate with nutrition specialists to design meal plans.

The other review article included in this Special Issue could be seen as an extension of the review conducted by Önal et al. [11]. Compañ-Gabucio et al. performed a scoping review [12] using a systematic methodology to describe dietary assessment tools used in intervention studies with children with ASD. They identified a total of thirteen different assessment tools among which The Brief Assessment scale for Mealtime Behavior in Children (BAMBI) and 24-hour recalls were the most frequently used, mainly due to the multifactorial nature of the first and ease of administration of the second. This scoping review revealed several knowledge gaps, including a clear need for more studies in Spain and across Europe, as well as studies with larger sample sizes and longer post-intervention follow-ups. We encourage readers of this Special Issue to explore new research initiatives to address these gaps.

Nutrition and eating behavior in children with NDDs are areas that require individualized attention and personalized strategies. It is essential to apply a holistic and integrative approach that combines nutrition with other therapies to help these children to deal with the multiple challenges that they face. A multidisciplinary approach involving nutritionists, occupational therapists, psychologists, teachers, and parents is needed to provide comprehensive support and significantly enhance the quality of life of children with NDDs and their families. In this regard, the studies in this Special Issue provide recent scientific evidence which forms the basis of the implementation and evaluation of effective interventions which can improve the health and functionality of these children on a global scale.
